# Renal insufficiency predicts worse prognosis in newly diagnosed IgD multiple myeloma patients

**DOI:** 10.3389/fonc.2022.1012889

**Published:** 2022-11-23

**Authors:** Ge Yan, Huangmin Li, Yiding Zhang, Chenyan Xia, Mengxiao Wang, Yu Jia, Jin Shang, Zhanzheng Zhao

**Affiliations:** ^1^ Department of Nephrology, The First Affiliated Hospital of Zhengzhou University, Zhengzhou, China; ^2^ School of Medicine, Zhengzhou University, Zhengzhou, Henan, China; ^3^ Laboratory of Nephrology, The First Affiliated Hospital of Zhengzhou University, Zhengzhou, Henan, China; ^4^ Laboratory Animal Platform of Academy of Medical Sciences, Zhengzhou University, Zhengzhou, Henan, China

**Keywords:** IgD multiple myeloma, renal function, cytogenetics, survival analysis, dialysis

## Abstract

**Objective:**

IgD multiple myeloma (MM) is a rare type of MM, accounting for about 1%–2% of all MMs. IgD MM always causes kidney damage and even leads to renal failure, which is the most common complication. This study aimed to explore the risk factors of renal damage and prognosis of IgD MM patients.

**Design:**

From March 2018 to November 2021, 85 patients with IgD MM diagnosed for the first time at the First Affiliated Hospital of Zhengzhou University were included in this study. We collected information on clinical features and laboratory examinations. Patients were divided into the renal impairment (RI) (47/85) and non-renal impairment (no-RI) (38/85) groups. Binary logistic regression was used to explore risk factors of renal damage. The Chi-square test was used to analyze the difference in chemotherapy effect between the two groups. We also analyzed whether early dialysis was beneficial to acute renal failure (RF) in IgD MM patients. Finally, Kaplan-Meier was used to compare the survival of the two groups.

**Results:**

In IgD MM, 55.3% of patients had renal damage as a complication, of which up to 59.6% presented with acute renal failure as the first manifestation. Serum β2-microglobulin (β2-MG) was an independent risk factor for renal damage in IgD MM (*p* = 0.002), but cytogenetic analysis suggested that it had no effect on patients’ renal damage. There was also no significant difference in the effect of chemotherapy between the two groups (*p* = 0.255). In patients with acute renal failure, there was no significant difference between dialysis and no dialysis groups in the proportion of patients with improved renal function after treatment. The median overall survival (OS) of the RI group was significantly shorter than that of the no-RI group (*p* = 0.042). In the RI group, the median OS was 29 months, and in the no-RI group, the median OS was > 40 months.

**Conclusion:**

Elevated serum β2-MG is an independent risk factor for renal damage. Compared with the no-RI group, patients in the RI group had poorer prognosis and shorter median OS. For patients with acute renal failure as the first manifestation, the treatment of primary disease is more meaningful than dialysis.

## Introduction

Multiple myeloma (MM) is a proliferative disease of malignant plasma cells, with its incidence taking the second place among hematological tumors (1–3). MM can be divided into different classes according to subtypes of secreted immunoglobulins. IgD MM accounts for 1%–2% of all MMs (2, 3). However, in China, studies have shown that the IgD MM proportion exceeds 5% (2, 4). MM treatment is a long-term process, which results in a great psychological burden to patients. Although studies have described clinical characteristics and prognosis of IgD MM, few people have paid attention to the characteristics of IgD myeloma patients with renal impairment in recent years (2). Our research mainly aimed at these patients.

There are many complications due to MM, including bone disease, anemia, renal insufficiency, and hypercalcemia, among which renal failure is the second most common cause of death. Studies have shown that the risk of renal damage in IgD MM is increasing (5), while the age of renal damage patients tends to be younger. During MM, more than 50% of patients suffer from renal damage. In some patients, acute renal injury is the initial manifestation (6).

There are multiple and complex cytogenetic abnormalities in clonal plasma cells of MM. In MM, cytogenetic abnormalities affect the progress, treatment response, and prognosis of the disease (7, 8). Whether specific chromosomal abnormalities lead to renal damage in some MM patients has also aroused interest.

This study aimed to explore the risk factors of renal damage in IgD MM, the effects of cytogenetic abnormalities on renal damage, and prognosis of these patients.

## Methods

### Study population and ethical approval

In this population-based retrospective analysis from March 2018 to November 2021, patients with positive serum IgD M protein were selected in the First Affiliated Hospital of Zhengzhou University. The inclusion criteria were as follows (1): age of 18–80 years (2), primary diagnosis of MM, and (3) positive serum IgD M protein. The exclusion criteria were (1) incomplete information and (2) having other primary glomerular diseases. Finally, a total of 85 patients were included. At the same time, in order to compare and explore the special clinical manifestations of renal damage in IgD MM. we randomly sampled 141 patients diagnosed with light chain MM from the patients with positive serum M protein during the same period of time, and collected patient related test results.

The First Affiliated Hospital of Zhengzhou University Ethics Review Committee granted ethical approval for the study, and the ethics review approval ID was “KY-2022-0531.”

### Grouping

According to the International Myeloma Working Group (IMWG) and the global organization for the prognosis of kidney disease (9–11), renal impairment criteria are defined as serum creatinine (Scr) of > 177 μmol/L. Accordingly, we divided patients into 47 cases of renal insufficiency (RI) group and 38 cases of normal renal function group (no-RI). Severe acute renal failure (RF) was defined as estimated glomerular filtration rate (eGFR) of < 15 ml/min/1.73 m^2^.

### Data collection

We collected the basic information and laboratory results of all patients. Basic information included age, sex, blood pressure, and blood glucose levels. Laboratory results included the proportion of plasma cells in bone marrow (BM), blood M protein content, red blood cell (RBC) count, white blood cell (WBC) count, platelet (PLT) count, hemoglobin (Hb) levels, serum potassium (K), serum calcium (Ca), blood urea nitrogen (BUN), blood uric acid (UA), serum creatinine (Scr), blood glucose (GLU), total cholesterol (TC), triglyceride (TG), albumin (ALB), β2-microglobulin (β2-MG), lactate dehydrogenase (LDH), estimated glomerular filtration rate (eGFR), C-reactive protein (CRP), erythrocyte sedimentation (ESR), and results of serum immunofixation electrophoresis. Additionally, we also collected the result of cytogenetics and the remission of MM after three cycles of chemotherapy. Then, we followed up on the survival of these patients. The deadline was May 2022.

### Efficacy assessment

According to the guidelines for the diagnosis and management of MM in China (12), efficacy criteria were divided into complete remission (CR), part remission (PR), disease stability (SD), and disease progression (PD). Improvement of renal function was defined as withdrawal from dialysis or more than a 50% decrease in creatinine in patients without dialysis. Failure of renal function was defined as a decrease in creatinine of < 50% in patients without dialysis, maintained dialysis state, or kidney transplant.

### Follow up

All patients were followed up until May 2022. Overall survival (OS) was defined as the time from the beginning of diagnosis to the end of death for any cause or the final follow-up.

### Statistical analysis

We compared the basic information of the two groups. The continuous variables were tested for normality. The categorical data were expressed as percentages. Mean ± standard deviation (SD) or median (quartile 1, quartile 3) was described as a continuous variable satisfying or not satisfying the normal distribution, respectively. Categorical variables were analyzed by Chi-square test. Continuous variables were analyzed by two independent sample t-test. Then, we conducted univariate and multivariate logistic regression analyses of the biochemical indexes. We analyzed whether there were differences in survival between the two groups using the Kaplan-Meier method. The relevant statistical analysis steps were performed by SPSS, version 25, and R software, version 4.1.2. A *P* of < 0.05 was considered statistically significant.

## Results

### Baseline characteristics

A total of 85 patients with IgD MM represented 1.6% of all MM patients. Among all 85 IgD MM patients, 53 (62.3%) were males, while 32 (37.6%) were females. The average age of these patients was 58.4 years, ranging from 27 to 82 years. λ light chain type (78/85) was the main immunoglobulin type, accounting for 91.7%. Forty-seven cases of the RI group accounted for 55.3% (47/85) of all patients.

As for each group, the characteristics showed certain differences. There were significant differences in the proportion of patients with hypertension between the two groups (*p* = 0.03). However, there was no significant difference in the average patients’ age, the proportion of males, and the proportion of diabetes patients. There were significant differences in the proportion of patients with abnormal serum tests, such as UA, ALB, β2-MG, and LDH, between the two groups. UA level in the RI group was higher than that in the no-RI group (*p* < 0.001). Hypoalbuminemia rate in the RI group was higher compared to the no-RI group (*p* = 0.001). More patients showed elevated serum β2-MG in the RI group compared to the no-RI group (*p* < 0.001). The proportion of patients with elevated LDH in the RI group was also higher (*p* = 0.014). There was no significant difference in M protein level, the number of bone lesions, the proportion of patients with elevated leukocytes, anemia, hypercalcemia, hypertriglyceridemia, and reduction of three immunoglobulins (**Table 1**).

**Table 1:** Baseline characteristics of RI and no-RI patients.

Characteristics		RI	no-RI	p-value
age (x ± s)		60 ± 11	56 ± 11	0.081
male/female		34/13	19/19	0.035
hypertension	no	24	31	
	yes	23	7	0.003
diabetes	no	42	35	
	yes	5	3	0.954
bone lesion		24 (55.8%)	22 (68.7%)	0.391
M protein (g/L)		4.75 (1.96, 13.78)	5.62 (2.27, 17.18)	0.854
WBC>9.5 (1012/L)		6 (12.8%)	1 (2.6%)	0.124
Hb <100 (g/L)	no	12	16	
	yes	35	22	0.106
Ca>2.7 (mmol/L)		8 (17%)	4 (10.5%)	0.393
UA (μmol/L)		508.94 ± 242.92	375.16 ± 142.05	<0.001
Scr (μmol/L)		562.33 ± 254.94	91.86 ± 31.71	<0.001
TG>1.7 (mmol/L)		20 (45.5%)	9 (26.5%)	0.085
ALB<35 (g/L)		11 (24.3%)	0 (0%)	0.001
β2-MG>8 (mg/L)		35 (89.7%)	7 (18.9%)	<0.001
LDH>245 (U/L)		26 (60.5%)	11 (32.4%)	0.014
triple hit		35 (100%)	29 (100%)	>0.999
κ-λ (mg/L)		4165.80 (708.80, 8930.80)	2707.00 (254.15, 4385.07)	0.002

BM, bone marrow; M protein, blood M protein content; WBC, white blood cell; Hb, hemoglobin; Ca, serum calcium; UA, uric acid; TG: triglyceride; ALB, albumin; β2-MG, β2-microglobulin; LDH, lactate dehydrogenase; triple hit, serum protein electrophoresis showed that IgA, IgG, and IgM decreased; free λ, serum dissociation λ light chain; κ-λ, serum dissociation κ light chain and λ light chain difference; Scr, serum creatinine.

### Serum β2-MG is an independent risk factor for renal damage

We first conducted a single-factor binary logistic regression analysis between the RI and no-RI groups. There was no significant difference in RBC, Hb, PLT, K, Ca, Scr, GLU, TC, CRP, ESR, M protein, free κ light chain, or κ/λ between the two groups (*p* > 0.05). There was a significant difference in WBC, BUN, UA, TG, ALB, β2-MG, LDH, eGFR, free λ light chain, and κ-λ between the two groups (*p* < 0.05, **Table 2**). Then, according to clinical knowledge, we deleted the factors related to grouped variables and included WBC, ALB, β2-MG, TG, LDH, and free λ light chain into the multifactor analysis. The results showed that there was no significant difference in WBC, ALB, TG, free λ light chain, and LDH between the two groups (*p* > 0.05). Only β2-MG difference was statistically significant (odds ratio [OR] = 1.753, 95% confidence interval [CI] 1.239-2.481, *P* = 0.002); thus, we considered β2-MG an independent risk factor of renal damage in IgD MM (**Table 2**).

**Table 2:** Potential risk factors identified by univariate and multivariate logistic regression analyses.

Characteristics	Univariable			Multivariable		
	OR	CI	p-value	OR	CI	p-value
WBC (10^12^/L)	1.418	1.115-1.804	0.004	1.302	0.749-2.263	0.349
RBC (10^9^/L)	0.687	0.402-1.174	0.170			
Hb (g/L)	0.989	0.972-1.007	0.240			
PIT (10^9^/L)	1.004	0.998-1.009	0.173			
K (mmol/L)	1.226	0.658-2.285	0.521			
Ca (mmol/L)	0.996	0.955-1.039	0.858			
BUN (mmol/L)	4.027	1.865-8.694	<0.001			
Scr (μmol/L)	5.389	0-2.418E+30	0.961			
UA (μmol/L)	1.007	1.003-1.010	<0.001			
GLU (mmol/L)	1.117	0.750-1.664	0.586			
TC (mmol/L)	1.296	0.914-1.837	0.145			
TG (mmol/L)	2.274	1.097-4.714	0.027	1.148	0.261-5.042	0.855
ALB (g/L)	0.918	0.847-0.993	0.034	0.885	0.707-1.108	0.286
β2-MG (mg/L)	1.576	1.287-1.932	<0.001	1.921	1.200-3.075	0.007
LDH (U/L)	1.003	1.000-1.006	0.048	1.002	0.994-1.009	0.652
eGFR (ml/min/1.73m^2^)	0.776	0.652-0.925	0.004			
CRP (mg/L)	1.008	0.991-1.025	0.377			
ESR (mm/h)	1.000	0.998-1.013	0.993			
M protein(g/L)	0.985	0.930-1.043	0.597			
free κ light chain (mg/L)	1.000	0.999-1.000	0.714			
free λ light chain (mg/L)	1.000	1.000-1.000	0.031	1.000	1.000-1.000	0.985
κ/λ	1.006	0.990-1.023	0.441			
κ-λ (mg/L)	1.000	1.000-1.000	0.019			

RBC, red blood cell; PLT, platelet; K, serum potassium; BUN, serum urea nitrogen; GLU, fasting blood glucose; TC, total cholesterol; eGFR, estimated glomerular filtration rate; CPR, C-reactive protein; ESR, erythrocyte sedimentation rate; free κ light chain, serum dissociation κ light chain; κ/λ, serum dissociation κ light chain and λ light chain ratio.

### Cytogenetics has no effect on renal damage in IgD MM

Fluorescence immune hybridization (FISH) examination of bone marrow biopsy was performed in 59 patients. In the RI group, FISH was performed in 33 patients, of which 24 patients (72.7%) had abnormal results. The incidence of positive 1q21 gene amplification was the highest, accounting for 54.5%. Other chromosomal abnormalities included positive *RB1* (13q14) gene deletion, accounting for 45.5%; positive *p53* (17p13.1) gene deletion, accounting for 6.1%; positive *D13S319* (13q14.3) gene deletion, accounting for 42.4%; and positive *IGH* (14q32) gene disruption, accounting for 48.5%. In the no-RI group, FISH was performed in 26 patients, of which 19 patients (73%) had abnormal results. Compared to the RI group, the abnormal proportion of positive *IGH* (14q32) gene disruption and positive 1q21 gene amplification were both very high, accounting for 50%. Other chromosomal abnormalities included positive *RB1* (13q14) gene deletion, accounting for 23.1%; positive *p53* (17p13.1) gene deletion, accounting for 7.7%; and positive *D13S319* (13q14.3) gene deletion, accounting for 23.1%. However, there was no difference between the RI and no-RI groups in FISH examination results (*p* > 0.05, **Table 3**). There were 15 patients with positive *IGH* (14q32) gene disruption and complete information (7 patients in the no-RI group and 8 patients in the RI group). For *IGHCCND1* gene positivity, there were 2 (28.6%) patients in the no-RI group and 5 (62.5%) persons in the RI group. Both groups showed no positivity for *IGHFGFR3* gene fusion and *IGHMAF* gene.

**Table 3:** Cytogenetic results of the RI and no-RI groups.

FISH	RI	no-RI	*p-*value
	(n=33)	(n=26)	
*RB1* (13q14)	15(45.5%)	6(23.1%)	0.075
*P53* (17p13.1)	2(6.1%)	2(7.7%)	>0.999
*D13S319* (13q14.3)	14(42.4%)	6(23.1%)	0.119
*IGH* (14q32)	16(48.5%)	13(50%)	0.908
1q21	18(54.5%)	13(50%)	0.728

### Hematological remission and renal function recovery after treatment

Of all patients, 53 patients had the follow-up results after four cycles of induction protocol. Patients received different bortezomib-based chemotherapy regimens. These regimens were bortezomib + dexamethasone, bortezomib + cyclophosphamide + dexamethasone, and bortezomib + lenalidomide + dexamethasone. For the bortezomib + dexamethasone regimen, there were 10 (41.4%) patients in the RI group and 10 (34.5%) patients in the no-RI group. For the bortezomib + cyclophosphamide + dexamethasone regimen, there were 7 (29.1%) patients in the RI group and 9 (31.0%) patients in the no-RI group. For the bortezomib + lenalidomide + dexamethasone regimen, there were 7 (29.1%) patients in the RI group and 10 (34.5%) patients in the no-RI group. There were 29 patients in the no-RI group and 24 patients in the RI group. The overall remission rate was 81.1%. The remission rate of the RI group was higher than that of the no-RI group. Unfortunately, there was no significant difference between the groups (*p* = 0.793, **Table 4**).

**Table 4:** Effects of chemotherapy in different groups on renal function impairment.

curative effect	no-RI	RI	Total
CR, PR	24 (82.8%)	19 (79.2%)	43 (81.1%)
SD, PD	5 (17.2%)	5 (20.8%)	10 (18.9%)
Total	29 (100%)	24 (100%)	53 (100%)

no-RI, normal renal function group; RI, renal function damage group; CR, complete remission; PR, part remission; SD, disease stability; PD, disease progression.

The baseline mean serum creatinine of patients with renal impairment due to IgD MM was 562.33 μmol/L, while that of patients with renal impairment due to light chain MM was 488.04 μmol/L (**Supplementary Table 1**). Compared with IgD MM, patients with light chain MM had lower mean serum creatinine, but there was no significant difference between the two groups (*p* = 0.152). After four cycles of chemotherapy with the bortezomib-based regimen, the improvement rate of renal function was 54.5% in the IgD MM renal damage group and 50.0% in the light chain MM renal damage group, but the difference was not statistically significant (*p* = 0.746) (**Supplementary Table 2**).

Some IgD MM patients with severe acute RF received emergency dialysis treatment. Dialysis lasted for about one chemotherapy cycle. If the patient’s renal function continued to decline, they required dialysis. Our analysis showed that in the dialysis group, the mean serum creatinine before dialysis was 803.83 μmol/L, and the mean serum creatinine after dialysis was 340.00 μmol/L. We compared the recovery of renal function between the dialysis and no-dialysis groups after four months of chemotherapy to explore whether early dialysis was conducive to the recovery of renal function. The results showed that there was no significant difference in the improvement of renal function, which might have been caused by our small sample size (**Table 5**). We also performed a similar analysis in patients with light chain MM renal damage. Among 141 patients with light chain MM, 46 patients with renal damage, accounting for 32.6%. Although the improvement rate of the dialysis group was higher than that of the no-dialysis group in light chain MM (50.0 *vs.* 62.5%), there was no statistical difference (*p* = 0.675) (**Supplementary Table 3**).

**Table 5:** Improvement of renal function in dialysis and no dialysis patients with IgD MM and acute RF.

curative effect	no-dialysis	dialysis	Total
Improvement	6(66.7%)	5(62.5%)	11(64.7%)
No Improvement	3(33.3%)	3(37.5%)	6(35.3%)
Total	9(100%)	8(100%)	17(100%)

### Survival analysis

**Figure 1:**
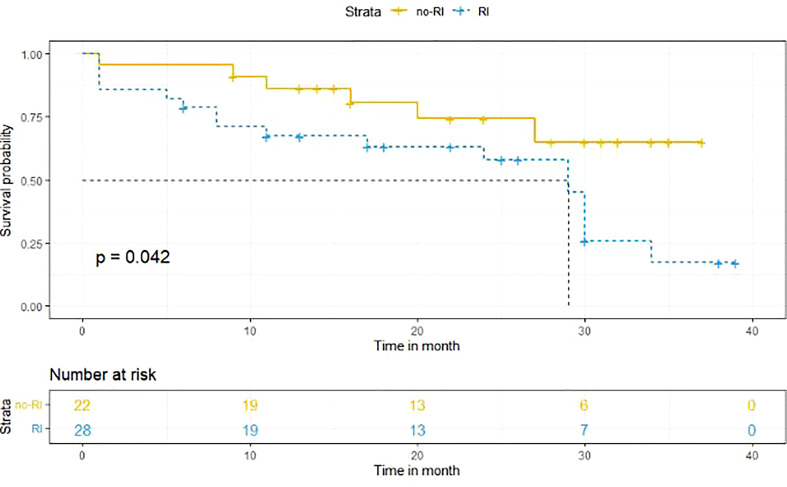
Survival analysis of the RI and no-RI groups. the median OS in the RI group was shorter than that in the no-RI group. The median OS in the RI group was 29 months, while the median OS in the no-RI group was > 40 months (*p* = 0.042).

Kaplan-Meier method was used to determine the survival difference between the two groups. Results suggested that the median OS in the RI group was shorter than that in the no-RI group. The median OS in the RI group was 29 months, while the median OS in the no-RI group was > 40 months. Due to insufficient sample size, the median survival time in the no-RI group could not be accurately obtained. The difference between the two groups was statistically significant (*p* = 0.042) (**Figure 1**).

## Discussion

From March 2018 to November 2021, 85 IgD MM patients diagnosed for the first time in the First Affiliated Hospital of Zhengzhou University were included in this study. Renal damage, as a complication, developed in 55.3% of patients, of which up to 59.6% had acute RF as the first manifestation. Our study described the relevant clinical characteristics of IgD MM patients with renal impairment and provided relevant reference materials. Moreover, our study showed that for patients with acute RF as the first manifestation, treatment of primary disease is the most important.

According to the literature, IgD MM currently develops at a younger age compared with other types of MM (4). The median onset age of IgD MM is 51–57 years. Male patients outnumber female patients, accounting for 62%–67%. The various subtypes based on serum free light chain analysis are divided into λ (80%–100%) and κ (3%–4%) (13, 14). In this study, there were 85 cases of IgD MM, with 53 males (62.3%) and 32 females (37.6%). The median age of onset was 58 years (27–82 years), and the λ light chain type was dominant, accounting for 91.7%.

Serum β2-MG is elevated in RF patients (1). Therefore, the increase in serum β2-MG in MM patients with RF is probably not only due to tumor burden. Perhaps, by measuring urinary and serum β2-MG together, we can distinguish whether increased serum β2-MG is due to poor renal function or tumor load. Some studies have shown that the increase in serum β2-MG and the decrease in albumin are indicators of poor prognosis in IgD MM patients (15). In this study, the value of serum β2-MG in the RI group was significantly higher than that in the no-RI group.

Ho et al. have analyzed data from 1069 MM patients. LDH of RI patients was significantly higher than that of no-RI patients (16). It has been confirmed by research that LDH reflects the tumor load (16). Therefore, researchers believe that high tumor load is a risk factor for renal damage in MM patients. In this study, the proportion of patients with elevated LDH in the RI group was higher than that in the no-RI group, but the results of multiple logistic regression analysis showed that LDH was not a risk factor for RI. This negative result might also be related to the small sample size.

Most patients with MM have genetic abnormalities. The cytogenetic changes detected by FISH are related to the patient’s prognosis. IGH translocation frequency in IgD MM patients was higher than that in other myeloma patients. According to a study on clinical manifestation and cytogenetics of MM in the Mayo clinic, MM with IgH rearrangement, especially t (14, 16), is more common in patients with high free light chain levels and renal function damage (17). However, in this study, there was no difference in IgH rearrangement between the RI and no-RI groups. The incidence of RI with positive chromosome 17 deletion detected during MM treatment has been reported to be higher in the RI group compared to the no-RI group, which were 12.5% and 6.9,% respectively (18). However, in this study, there was no difference between the RI and no-RI groups. This result might be due to insufficient sample size, or that poor prognosis of renal damage in IgD MM is not caused by cytogenetic abnormalities but is related to other reasons, such as abnormal biochemical indicators.

It has been confirmed that light chain MM and IgD MM are the two types most prone to renal function damage (19). Although our data showed that there was no significant difference in the severity of renal function damage between the two types, IgD MM showed a more serious trend of renal function damage. Perhaps, after expanding the sample size, there will be a difference. Normally, excessive serum free light chains are filtered through the glomerulus and then absorbed in the proximal tubule. However, when serum free light chains exceed the absorption capacity of the proximal tubule, these will cause damage to the proximal tubule cells. Therefore, the key to treating renal damage is to remove the excessive serum free light chain (1). European studies have shown that the removal of free light chains by prolonged dialysis has no benefit to patients during the follow-up, although these results might be partly attributed to the different chemotherapy regimens of these patients (20). On the other hand, some studies have also shown that rapid identification of myeloma nephropathy is a key factor for the recovery of renal function (21). Delayed diagnosis has been associated with lower survival (22). In our study, the renal function of IgD MM patients with RF did not improve by dialysis, which is consistent with the literature. In our opinion, for patients with acute RF as the first manifestation, the treatment of primary disease is more meaningful than dialysis.

Wang et al. have demonstrated that the median survival of 68 IgD MM patients was 24 months, while that of Korean and Chinese patients was shorter than that of European patients (14). In 2014, Zagouri et al. reported that the median OS of 31 IgD MM patients in Greece was 51.5 months, making them the oldest and longest surviving IgD patients reported thus far (23). RF, especially RF requiring continuous dialysis, is a predictor of low survival in MM patients (24). In this study, the median OS in the no-RI group was > 40 months, and the median OS in the RI group was 29 months. With the improvement of treatment, the median OS of such patients has been significantly longer than before.

Our study aimed at the specific population with renal damage caused by IgD MM. Only a few people have done such research before. Our study was limited by the retrospective design and single-center data. Additionally, the small sample size limited the research results, which was also determined by the low incidence rate of the disease.

## Conclusion

Elevated serum β2-MG is an independent risk factor for renal damage due to IgD MM. Different cytogenetic results do not allow physicians to predict whether IgD MM will cause renal damage. Patients with RF as the first manifestation should be screened as soon as possible to see whether it is related to MM. Additionally, for these patients, treatment of primary disease is more meaningful than dialysis. Compared with the no-RI group, patients in the RI group had poorer prognosis and shorter median survival. Therefore, the management of IgD MM patients with renal impairment should be improved.

## Data availability statement

The original contributions presented in the study are included in the article/**Supplementary Material**. Further inquiries can be directed to the corresponding authors.

## Ethics statement

The studies involving human participants were reviewed and approved by The First Affiliated Hospital of Zhengzhou University Ethics Review Committee. Written informed consent for participation was not required for this study in accordance with the national legislation and the institutional requirements. Written informed consent was not obtained from the individual(s) for the publication of any potentially identifiable images or data included in this article.

## Author contributions

ZZ and JS provided financial support. JS helped to design this study. GY and YJ collected clinical data for this project. GY, MW, CX, YZ, and HL completed the draft of this manuscript. JS supervised the study and revised the manuscript. All authors contributed to the article and approved the submitted version.

## Funding

This work was supported by the National Natural Science Foundation of China (Grant Nos. 81873611 and 82170738) and the 2020 Key Project of Medical Science and Technology to JS.

## Acknowledgments

We thank all the participants who contributed to the article and the patients who provided the data.

## Conflict of interest

The authors declare that the research was conducted in the absence of any commercial or financial relationships that could be construed as a potential conflict of interest.

## Publisher’s note

All claims expressed in this article are solely those of the authors and do not necessarily represent those of their affiliated organizations, or those of the publisher, the editors and the reviewers. Any product that may be evaluated in this article, or claim that may be made by its manufacturer, is not guaranteed or endorsed by the publisher.
