# Supplementary material for: Renal insufficiency predicts worse prognosis in newly diagnosed IgD multiple myeloma patients
Source: Front Oncol. 2022 Nov 23;12:1012889. doi: 10.3389/fonc.2022.1012889 (PMC9727151; doi:10.3389/fonc.2022.1012889)
Supplement: Supplementary file 1 [file DataSheet_1.pdf]

Supplementary material

Supplementary Table 1. Baseline characteristics of IgD MM RI and light chain MM RI.

characteristics	IgD MM RI	Light chain MM RI	p-value
age (x±s)	60.28 ± 10.68	59.15 ± 9.52	0.593
male/female	34/13	31/15	0.608
κ-λ (mg/L)	4165.80 (708.80, 8930.80)	9435.6 (2531.35, 16905.80)	0.076
% of plasma cells in BM	34.91 ± 21.91	43.00 ± 34.91	0.131
Scr (μmol/L)	562.33 ± 254.94	488.04 ± 240.89	0.152
eGFR	11.93 ± 8.22	12.64 ± 7.37	0.666
Chemotherapy regimen			
BD	9	16	
VCD	7	10	
VRD	6	4	

Supplementary Table 2. Improvement of renal function in IgD MM RI and light chain MM RI.

curative effect	IgD MM RI	Light chain MM RI	Total number
renal function improved	12 (54.5%)	15 (50.0%)	27 (51.92%)
renal function no-improved	10 (45.4%)	15 (50.0%)	25 (48.08%)
Total number	22 (100%)	30 (100%)	52 (100%)

Supplementary Table 3. Improvement of renal function in dialysis and no dialysis patients with light chain MM and acute renal failure.

curative effect	no-dialysis	dialysis	Total
Improvement	7(50.0%)	5(62.5%)	12 (54.5%)
no-Improvement	7(50.0%)	3(37.5%)	10 (45.4%)
Total	14(100%)	8(100%)	22 (100%)
